# Trends in the direction of global plant invasion biology research over the past two decades

**DOI:** 10.1002/ece3.9690

**Published:** 2023-01-18

**Authors:** Jing Hua Chiu, Kwek Yan Chong, Shawn K. Y. Lum, David A. Wardle

**Affiliations:** ^1^ Asian School of the Environment Nanyang Technological University Singapore Singapore; ^2^ Singapore Botanic Gardens National Parks Board Singapore Singapore

**Keywords:** invasion biology, invasive plants, plants, plant ecology

## Abstract

Invasive plants are a growing ecological problem worldwide, but biases and patterns within invasive plant research may affect our understanding of invasive plant ecology. In this study, we analyzed 458 invasive plant papers sampled from the two journals dedicated entirely to the field of invasion biology, i.e., *Biological Invasions* and *Neobiota*. From these papers, we collected information on geographic coverage, climate, habitat, taxonomic coverage, plant functional type, and research topic to examine trends across a 21‐year time period from 1999 to 2020. Our analysis found that invasive plant research was consistently biased toward temperate grassland and forest ecosystems particularly within the Americas, Europe, and Australia, and toward smaller, herbaceous invasive plant species (i.e., forbs, grasses, and shrubs), with an increase in interest in invasive nitrogen‐fixing legumes over time. Our analysis also identified “hot” research topics in invasive plant research at specific time periods, such as a peak in the use of genetic analysis methods in 2014–2015 and a more recent focus on plant physiological and functional traits. While current models, concepts, and understanding of plant invasion ecology are still driven by such biases, this has been partially offset by recent increased research in understudied systems, as well as increasing awareness that plant invasion is heavily affected by their growth types, physiological traits, and soil interactions. As the field of invasion biology becomes ever increasingly important over time, focusing invasive plant research on understudied ecosystems and plant groups will allow us to develop a more holistic understanding of the ecology of invasive plants. In particular, given the outsized importance of the tropics to global biodiversity, the threats they face, and the dearth of studies, it is of critical importance that more invasive plant research is conducted within the tropics to develop a more globally representative understanding of invasive plant ecology.

## Graphical abstract

We analyzed 458 invasive plant papers sampled from two journals of invasion biology, *Biological Invasions* and *Neobiota*, across the past two decades. The analysis identified several trends and biases in invasion plant, and these findings help identify critically understudied areas of invasion plant biology worldwide, in particular within tropical ecosystems.

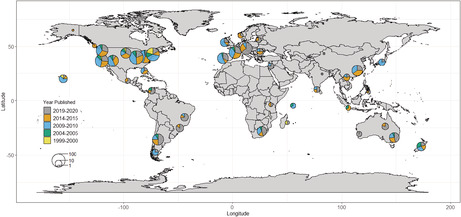

## 1 INTRODUCTION

**FIGURE 1:**
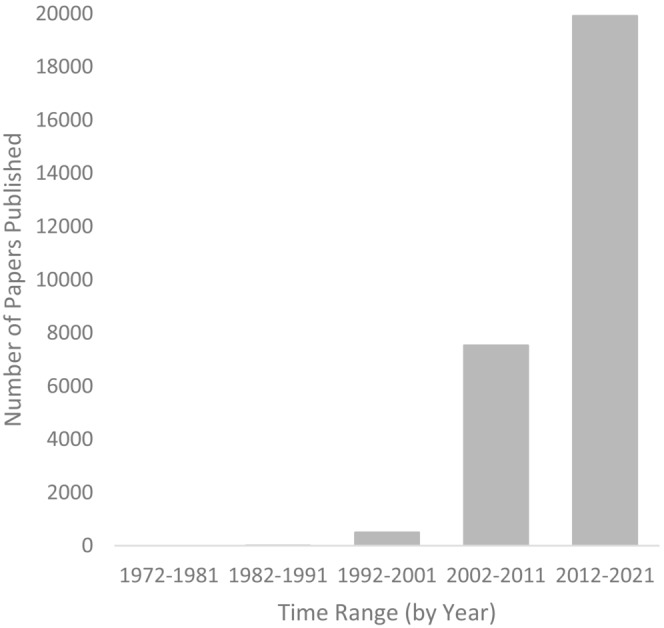
Number of invasive species papers published within the *Web of Science* (https://www.webofscience.com) over time. Search terms used (all within topic) were: “invasion biology” OR “invasive species” OR “biological invasion” OR invasive+ecology OR invasion+ecology.

Invasive species are an increasing ecological problem worldwide and can alter the diversity and composition of native ecosystems in a detrimental manner (Bellard et al., 2016; Murphy & Romanuk, 2014; Simberloff et al., 2013). Once established, invasive species often become difficult to remove, and their effects on native ecosystems are also often difficult to reverse (Kettenring & Adams, 2011; Mack & Lonsdale, 2002). Globalization has also increased opportunities for invasive species to be introduced to new ranges, and invasion is projected to be an increasing ecological problem worldwide (Seebens et al., 2015, 2021). Invasion biology (the study of invasive species), as an independent field of research in ecology, is a relatively young field (Simberloff et al., 2013). There has been a recent explosion of interest over the past 10–15 years in this topic (Chong et al., 2021; Figure 1), which now has two major journals dedicated exclusively to it—*Biological Invasions*, which started in 1999 (Carlton, 1999) and *Neobiota*, which started in 2011 (Kühn et al., 2011).

While invasive plant research is well represented in the general area of invasion biology (Pyšek et al., 2008), key areas within invasive plant research may be greatly underrepresented. Typical of many fields in ecology, the literature has historically been dominated by wealthier and developed countries (i.e., the Global North: America, Europe, Australia, and New Zealand), with a strong resultant bias in temperate ecosystems (Chong et al., 2021; Nuñez et al., 2022; Pyšek et al., 2008; Weidlich et al., 2020). As such, a recent analysis by Nuñez et al. (2022) found that a vast majority of all papers published within *Biological Invasions* were published by USA or European‐based scientists. Correspondingly, there is relatively widespread awareness of the harms that invasive plants can do in the temperate zone. “Classic” plant invasion examples that frequently attract attention and are frequently taught in university courses—for example, pines (*Pinus* spp.) in the temperate Southern Hemisphere, and kudzu (*Pueraria montana*) and the grass *Bromus tectorum* in North America—reflect such temperate biases in global recognition of invasive plant issues. Conversely, there have been few studies in tropical regions, except for particular island systems such as Hawaii (Chong et al., 2021; Weidlich et al., 2020), resulting in lower awareness of invasive plant issues in tropical areas. This is a significant problem, as tropical ecosystems contain much of the world's biodiversity (Barlow et al., 2018) and contain some of the world's most threatened ecosystems (Gardner et al., 2009; Laurance, 2013).

Furthermore, there may be other forms of biases and patterns within invasive plant research. As in all ecological research, fieldwork is important in invasive plant research. However, due to the inherent logistical difficulties of fieldwork, invasive plant research may be concentrated around easy‐to‐study and local plants and field sites. For instance, there may be a bias toward easily accessed ecosystems like grasslands and some types of forest (Weidlich et al., 2020). As an example, some of the more well‐known research coming from the Americas (both North and South) in invasive plants look at grasslands—whether it be invasion of various types of plants in the South American *Cerrado* Savanna (Pivello et al., 1999), or invasion of the grass *Bromus tectorum* into North American grasslands (Vitousek et al., 1997). Furthermore, researchers may tend to prefer to study ecosystems which are more local or familiar to them, which may contribute to such biases. In particular, Nuñez et al. (2022) found that that most of the invasion biology manuscripts that were submitted, reviewed, or accepted for the journal “*Biological Invasions*” arose from wealthier temperate regions, such the North America, Europe, and Australasia. There may also be biases in the types of plants being studied (Weidlich et al., 2020). In particular, certain taxonomic and functional groups of plants may also receive more attention due to being seen as more likely to become invasive. For instance, nitrogen fixers (most notably legumes within the Fabaceae) are widely recognized as powerful invaders (Daehler, 1998) because of their mutualisms with nitrogen‐fixing bacteria (Richardson et al., 2000), and research into invasive plants has the potential to pay disproportionate attention to this group. Finally, there may be biases in the topics and types of questions that are being studied within invasive plant research, and these could potentially change across time in a manner that follows the trends in ecological research as a whole. Two notable examples within the literature are a relatively recent increase of interest in applying genomic techniques to answering questions about invasion biology (Chown et al., 2015) and an increasing recognition of the importance of various plant physiological and functional traits in invasive plant success (Drenovsky et al., 2012).

Literature biases are a problem that needs to be examined in detail in any field of scientific research—such biases can impact on the theories, the outcomes of meta‐analyses and syntheses, and our fundamental views of any field. For example, substantive geographic bias of field sites has been shown to be a serious issue in ecological research in the Arctic (Metcalfe et al., 2018), studies on how food webs respond to global change (Cameron et al., 2019), and work on the relationship between biodiversity and ecosystem functioning (Clarke et al., 2017). Such biases may also prevalent in the field of invasion biology (Chong et al., 2021; Nuñez et al., 2022; Pyšek et al., 2008), and as a relatively novel field of research, it is of interest as to whether these biases have changed over time in this rapidly expanding research area. In this paper, we look at the papers published within the two major journals devoted exclusively to publishing on invasion biology, i.e., *Biological Invasions* (launched in 1999) and *Neobiota* (launched in 2011), from 1999 to 2020. Specifically, we aim to answer the following questions about what has been the focus of invasion research on invasive plants in invasion biology, and how these have changed across the 21 years since invasion biology really took off as a field and *Biological Invasions* was first launched:

1. What is the geographical distribution of research on invasive plants and how has this distribution changed over time?
2. What type of ecosystems or biomes do invasion ecologists study invasive plants in and has this changed over time?
3. What are the types of invasive plants that are being studied and are most studies focused on a handful of well‐studied species?
4. What sorts of questions do ecologists seek to answer about invasive plants and have these changed over time?

By addressing these questions, we aim to offer insights on how research foci and questions change over time in a rapidly developing and growing field of study. Some previous studies have considered the geographic biases of studies on invasion biology (Chong et al., 2021; Nuñez et al., 2022; Pyšek et al., 2008), but how these biases have changed over time, or other biases in invasion biology (e.g., taxonomic bias, or the types of questions that are being most commonly addressed) remain largely unstudied. The field of invasion biology will only become more important over time as the signature of human activity on the Earth's ecosystems becomes ever more apparent. Understanding the patterns of how invasive plant research has changed over the past two decades can help identify critical knowledge gaps and biases and inform and help predict future research directions within the field.

## 2 METHODS

All papers published between 1999 and 2020 in the journals *Biological Invasions* and *Neobiota* were sampled in 5‐year steps, with each step spanning two full years (i.e., 1999–2000, 2004–2005, 2009–2010, 2014–2015, 2019–2020). Papers were sampled in 5‐year steps because sampling every published paper across the two journals at the level of detail that we examined them would be intractable, and this provided enough separate steps (i.e., five) to meaningfully look at trends across this time period. From these papers, all papers involving collection of original data from field experiments, greenhouse (or similar) experiments, or observational studies of invasive plants, and excluding literature reviews, meta‐analyses, theoretical modeling studies, or studies with a primarily sociological focus, were selected for analysis, with a total of 458 papers selected in total (See Table 1 for breakdown of number of papers by year). The papers were read to ensure they satisfy these criteria, and details about them were tabulated. Observed trends were analyzed by fitting the category of paper as a logistic regression against time, and the statistical significance of the slope was assessed with a chi‐squared test. All analyses were conducted using R v3.6.3 (R Core Team, 2020). Data corresponding to each of the four questions were tabulated as follows:

**TABLE 1:** Number of papers analyzed in this study, sorted by year and journal

Journal	Year									
	1999	2000	2004	2005	2009	2010	2014	2015	2019	2020
*Neobiota*	0	0	0	0	0	0	2	1	16	13
*Biological Invasions*	12	4	15	20	47	100	56	58	81	65

### 2.1 Geographical distribution

Coordinates and location/region details of the location of each published study were collected as follows. First, the field site(s)/collection source(s) where the invasive plants were found or grown in the study were recorded. If exact co‐ordinates of the locations described above were available within the paper or its supplementary documentation, these co‐ordinates were converted to decimal degrees using the WGS84 datum (if necessary). If no co‐ordinates were available within the paper, but a map of the sites were provided, then approximate co‐ordinates were obtained by viewing the same area on Google Maps and recording the co‐ordinates of the area indicated by the map. If no co‐ordinates/maps were available, or if there were too many (>50) co‐ordinates indicated on a map, a single co‐ordinate positioned in the center of the study area was used. A cluster analysis was performed with the Euclidean distances between all pairs of location co‐ordinates, and the resulting dendrogram was cut to produce 45 unique clusters of locations. The latitude and longitude of the centroid of each cluster were then calculated from all the locations in that cluster and plotted onto a world map. Location data were also categorized into the following categories: Africa, Asia, Australia, New Zealand, Europe, North America, South America, Oceanic Islands, and Others. The number of studies per location was then plotted against time in 5 year steps. As studies may contain more than one location being studied, only unique paper location/co‐ordinates combinations were analyzed/plotted.

### 2.2 Climatic zone and ecosystem type

Climate and ecosystem type for each paper was either taken directly from the papers or inferred based on the location data. Climate data were categorized with the Köppen climate classification system and simplified by subsuming the Arid, Semi‐Arid & Desert climates into the Arid category; the Boreal, Subpolar, & Alpine climates into the Cold Weather category; and the Oceanic & Continental climates into the Temperate category. Each paper was also classified into the following categories of ecosystem type: Anthropogenic (Urban, Agricultural), Forest, Grasslands/Savanna, Wetlands (Riparian, Marshes, Swamps, and other wetlands), and Others (Shrublands, Sand Dune, and other ecosystems). The number of studies in each climate category and ecosystem‐type category were plotted against time in 5‐year steps. As studies may contain more than one climatic or ecosystem type being studied, unique paper climate/ecosystem combinations were taken as separate data points.

### 2.3 Plant taxonomic and functional identity

For this analysis, we looked only at papers studying specific invasive plant taxa, excluding observational community‐level or survey studies. The taxonomic identity of the plant species that were studied was collected directly from each paper, and taxonomic synonyms were resolved by using the species name most commonly accepted within the scientific community for that species. Plant species in each paper were also assigned to broad functional types using the USDA plant database, with the following commonly accepted categories: Forb, Graminoid, Shrub, Tree, Vine, and (nitrogen‐fixing) Legumes. Nitrogen‐fixing legumes were separated out as their own functional type because of the major role of this group as an ecosystem driver. The number of studies in each taxonomic order and functional type were plotted against time. As studies may contain more than one species or functional type being studied, only unique paper–species/functional type combinations were plotted. Unique paper–species combinations were used to account for different invasive plant species within the same order in the same paper.

### 2.4 Types of topics being studied

The types of topics being studied in each paper were determined by reading the abstracts and contents of the papers and broadly classified into the following subcategories: Invader Establishment, Invader Spread & Dispersal, Invader Plant Traits (Physiological), Invader Plant Traits (Genetic), Invader Impacts on Native Plant Community, Invader Impacts on Soil/Environment, Invader Control and Management, and Others. The number of studies belonging to each type was tabulated against time in 5‐year steps. As some studies answered more than a single type of question, only unique paper‐question combinations were tabulated.

## 3 RESULTS

### 3.1 Geographical distribution

**FIGURE 2:**
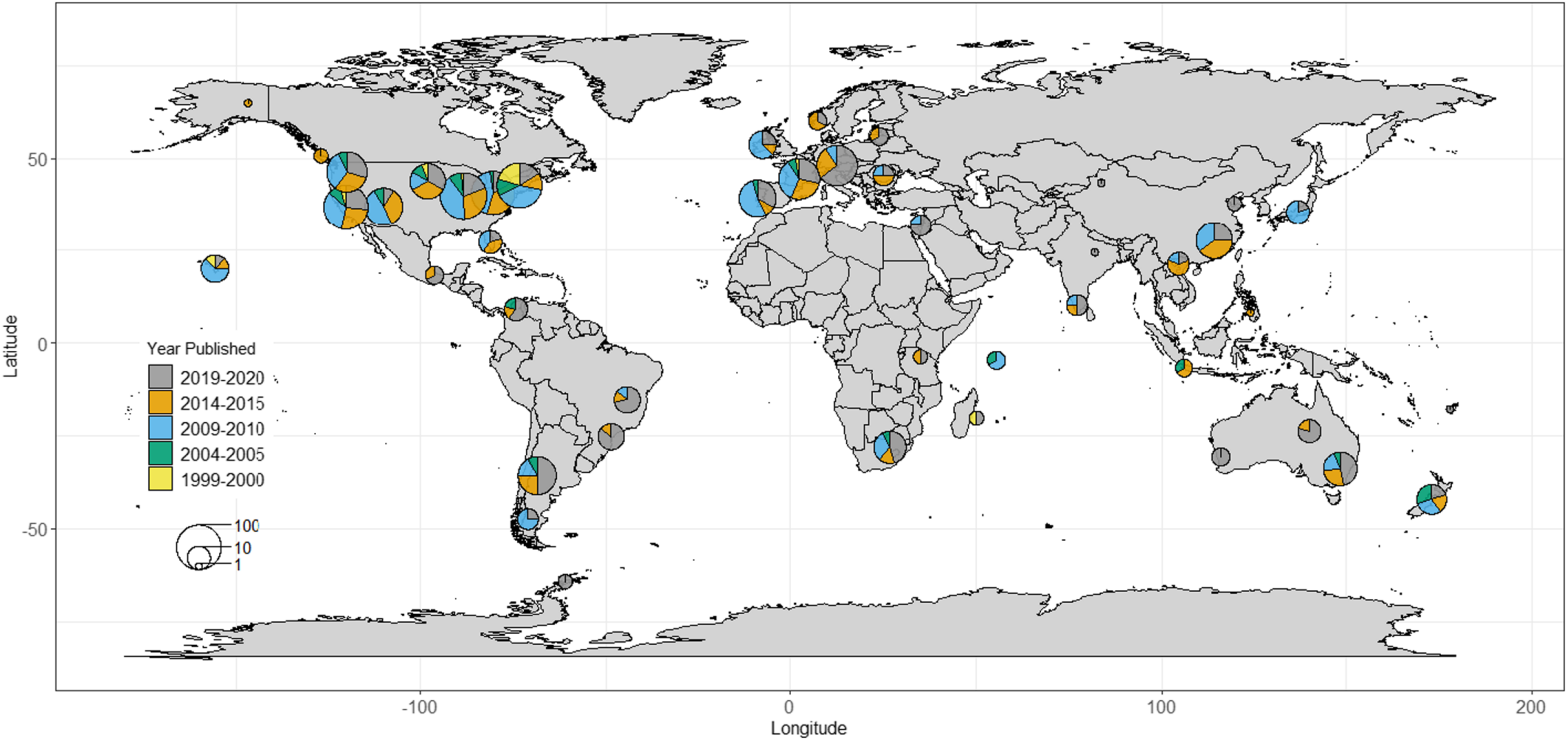
Geographical distribution of invasive plant studies published in *Biological Invasions* and *Neobiota* from 2009 to 2020, separated into five‐yearly time steps. The sizes of pie charts are proportional to the number of unique studies, while colors indicate the years that the studies were published.

**FIGURE 3:**
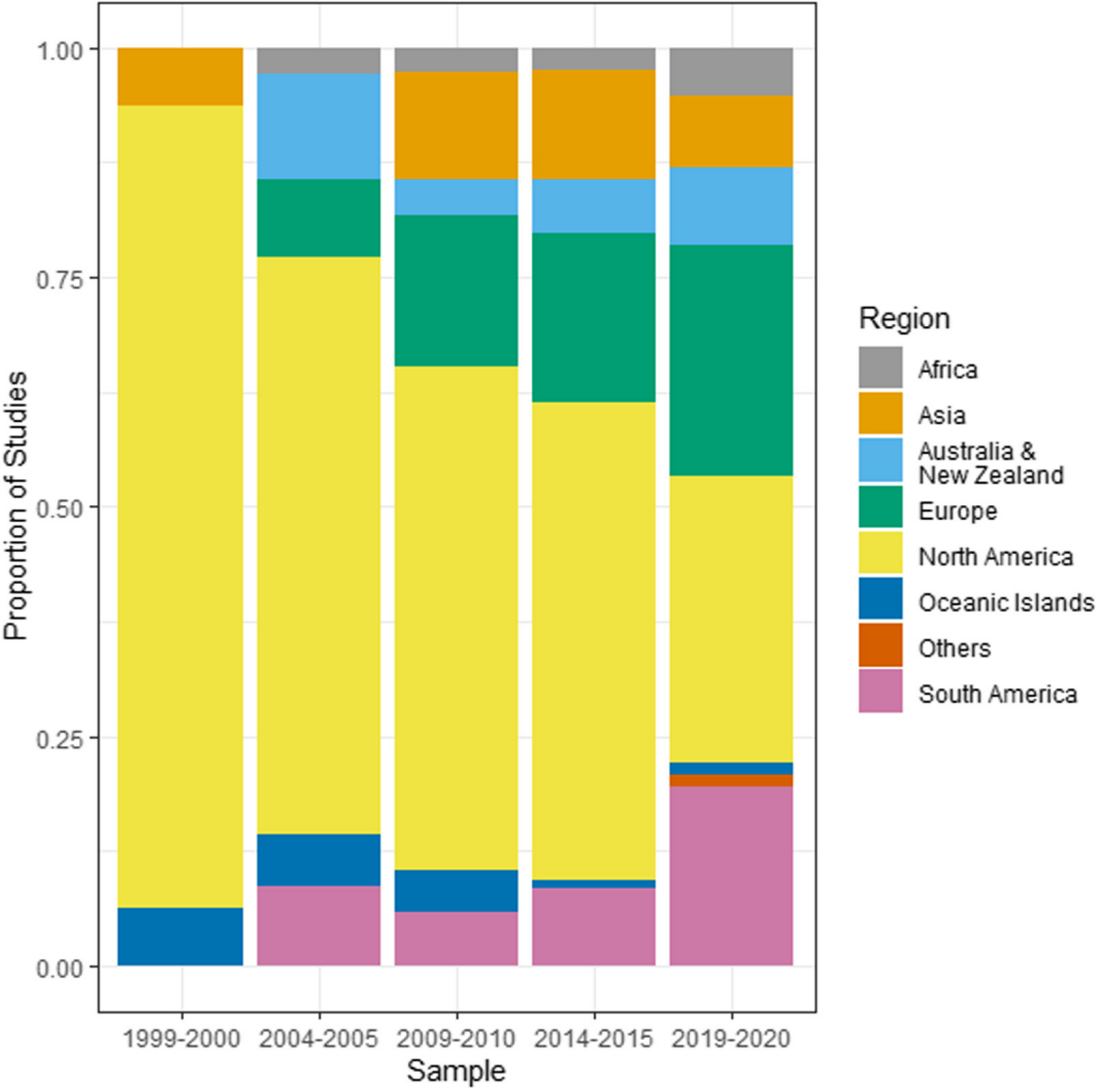
Regional distribution of invasive plant studies published in *Biological Invasions* and *Neobiota* from 1999 to 2020, showing proportion of studies published.

The vast majority (comprising approximately 83%) of studies have been conducted in the Americas and Europe, with smaller clusters of studies concentrated in Hawaii, South Africa, Australia, New Zealand, and China (Figure 2). Of note is the relative lack of studies conducted within the Tropics, except for Hawaii. Further, regional trends (Figure 3) show that the total number of studies within wealthier regions of the world (Americas, Europe, Australia/New Zealand) has consistently outnumbered the studies conducted in other parts of the world (in particular, Africa and parts of Asia) across time. Consistently, the highest number and proportion of studies are conducted in North America, with the number and proportion of studies in Europe and South America catching up over time (Europe: *χ* ^2^ = 11.0, df = 1, *p* < .001; South America: *χ* ^2^ = 15.6, df = 1, *p* < .001). The other regions show generally smaller, and mostly statistically significant increases over the recent years (Asia: *χ* ^2^ = 12.7, df = 1, *p* < .001; Australia/New Zealand: *χ* ^2^ = 44.2, df = 1, *p* < .001; Africa: *χ* ^2^ = 9.9, df = 1, *p* = .002; Oceanic Islands: *χ* ^2^ < 0.1, df = 1, *p* = .986), with the exception of a slight dip in Asian studies from 2009/2010 to 2019/2020.

### 3.2 Climatic zone and ecosystem type

**FIGURE 4:**
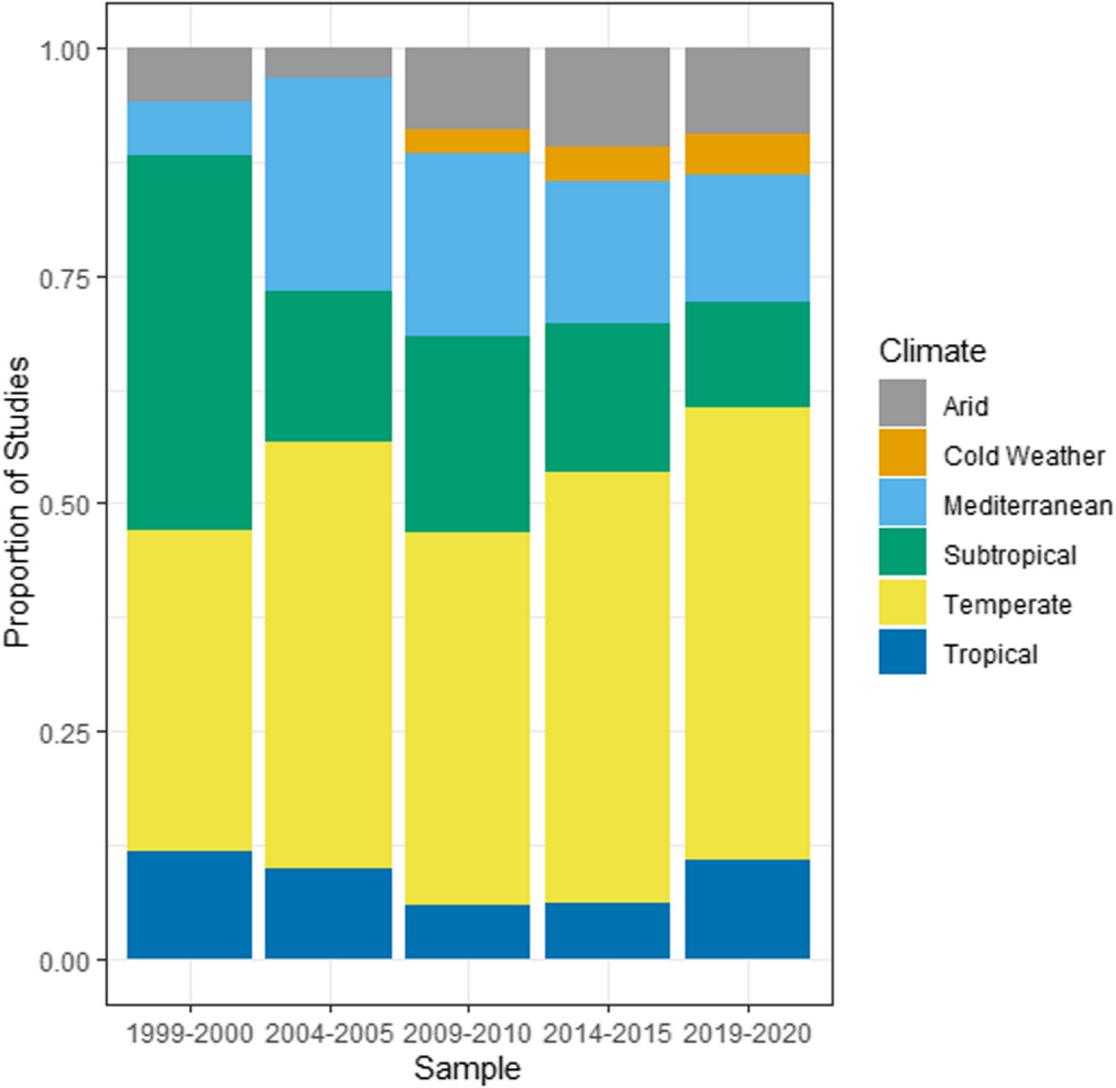
Invasive plant studies published in *Biological Invasions* and *Neobiota* from 1999 to 2020 categorized according to climate, showing proportion of studies published.

**FIGURE 5:**
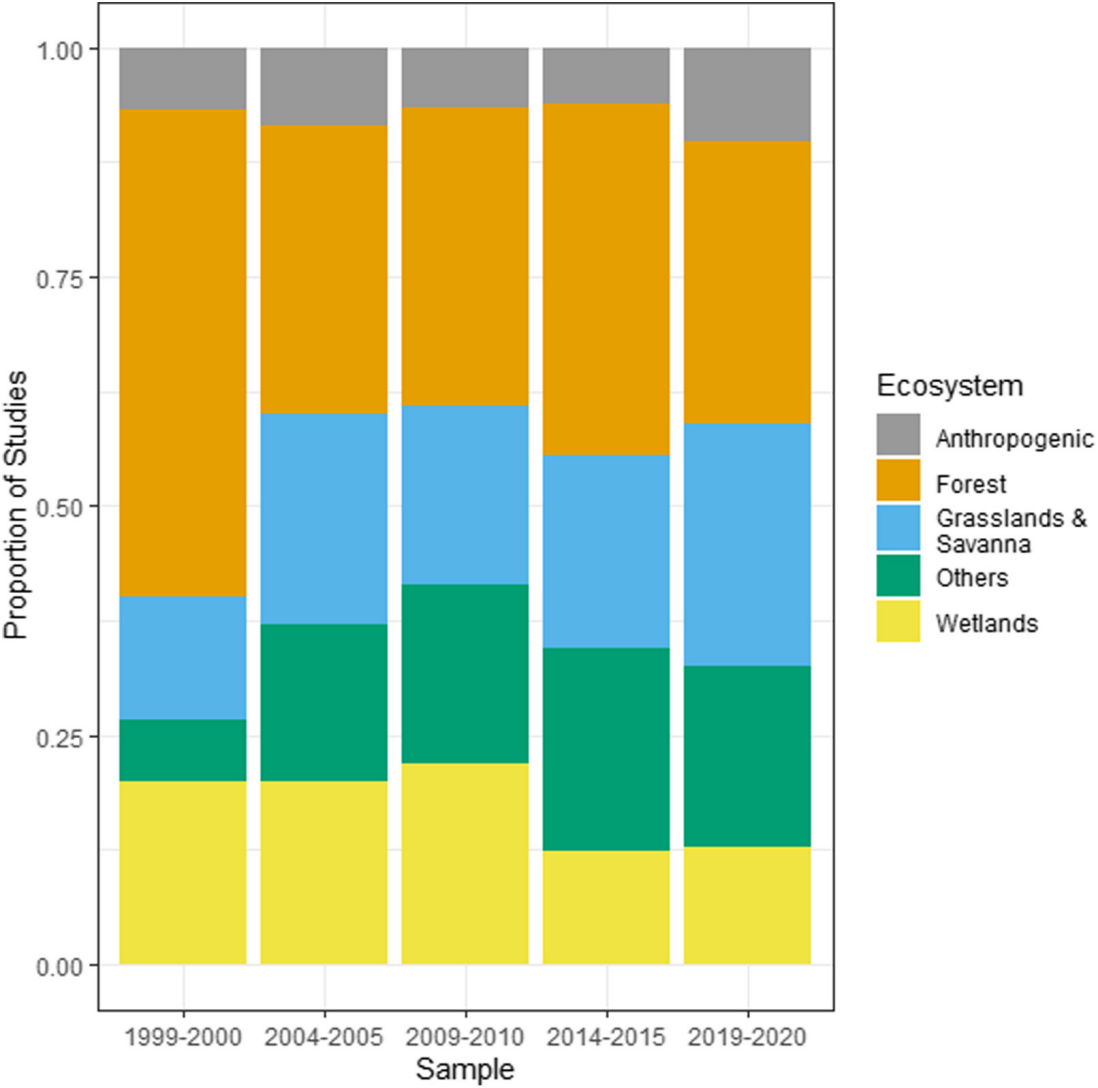
Ecosystems studied within invasive plant studies published in *Biological Invasions* and *Neobiota* from 1999 to 2020, showing proportion of studies published.

Invasive plant research is heavily concentrated within the Temperate, Mediterranean, and Subtropical climate regions across time (Figure 4), with the number of Temperate studies consistently being the highest. Other climate types showed sharp increases in number of studies in 2009/2010, due partially to a large increase in the number of total papers being published in that time period. However, the number of papers looking at Mediterranean, Subtropical, and Arid habitat types have flattened out since then, and the Tropical and Cold Weather habitats have shown small recent increases in studies being published from 2014/2015 to 2019/2020. The proportions of papers across different climate regions have not changed substantially over time (*p* > .05) other than a fall for subtropical studies from 2004/2005 onward (*χ* ^2^ = 8.1, df = 1, *p* = .004), with the highest proportion of studies in Temperate, Mediterranean, and Subtropical regions. In terms of ecosystems, the majority of invasive plant research has been conducted within forests and grasslands (Figure 5), and with the proportion of grassland studies increasing and forest studies decreasing from 1999/2000 to 2004/2005. Studies in wetlands and anthropogenic habitats have also increased over time from 1999/2000 to 2009/2010 and has stabilized thereafter.

### 3.3 Plant taxonomic and functional identity

**FIGURE 6:**
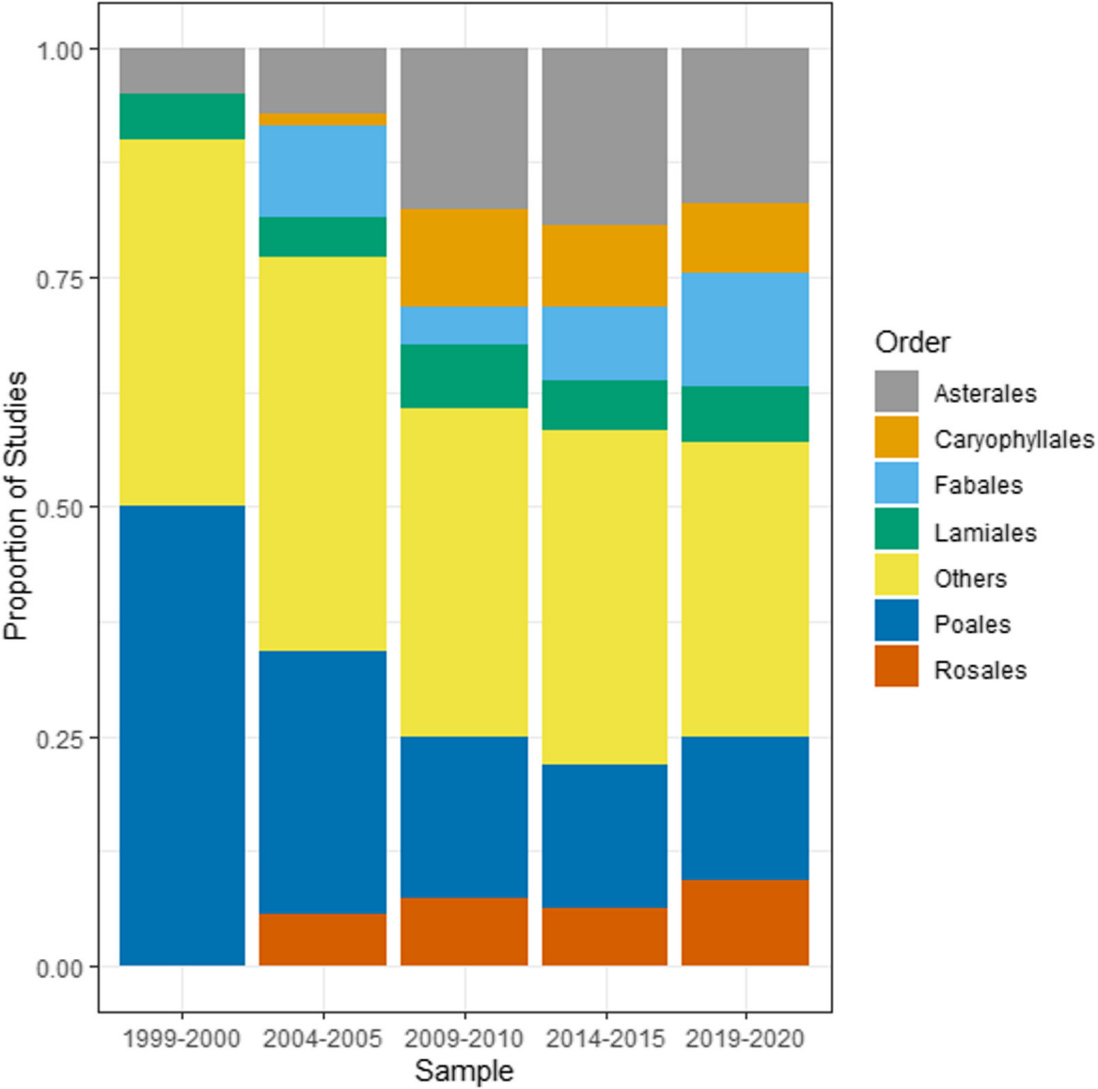
Invasive plant orders studied within invasive plant studies published in *Biological Invasions* and *Neobiota* from 1999 to 2020 showing proportion of studies published. The subcategory “Others” represents a total of 28 other orders.

**FIGURE 7:**
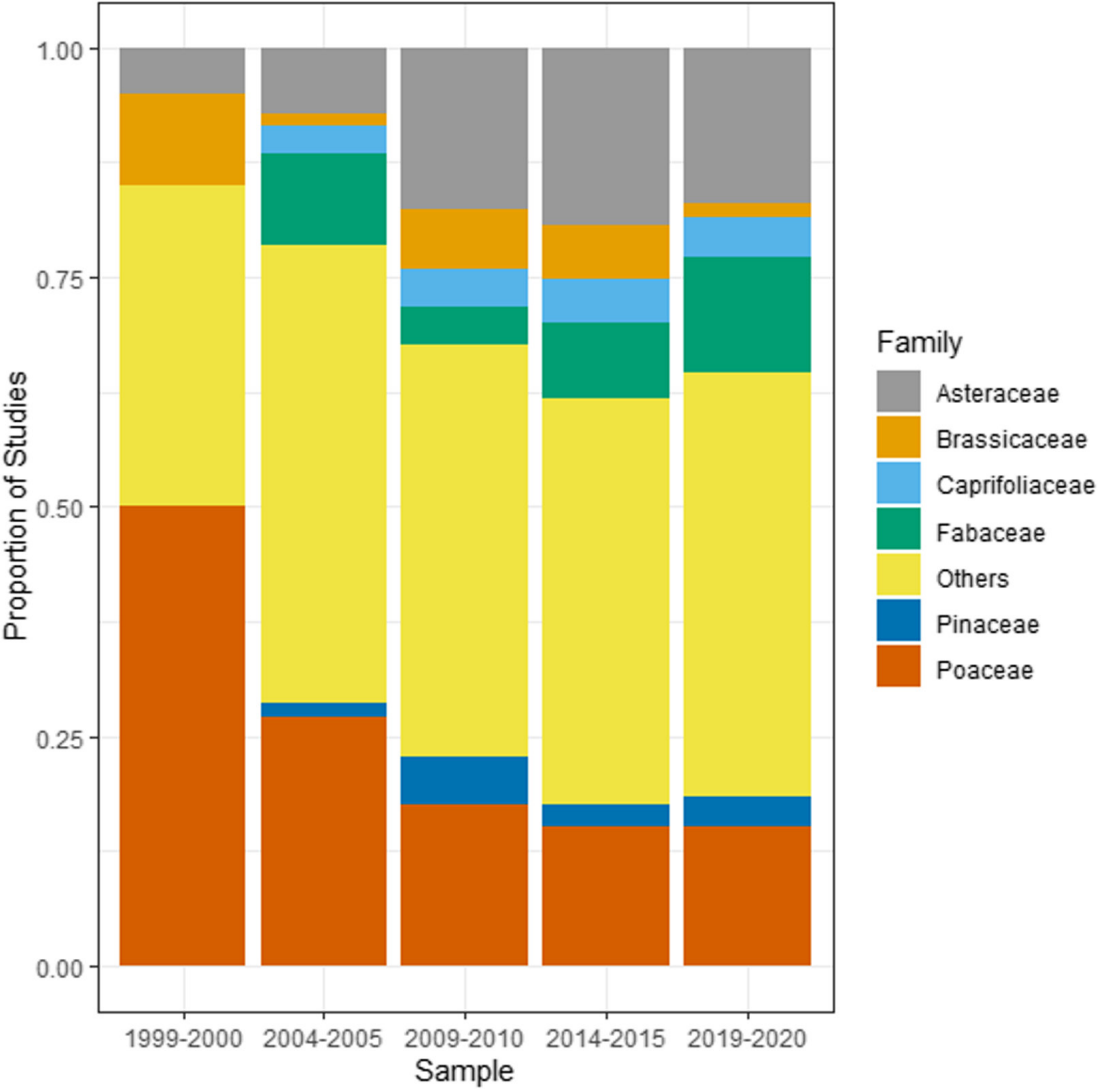
Invasive plant families studied within invasive plant studies published in *Biological Invasions* and *Neobiota* from 1999 to 2020, showing proportion of studies published. The subcategory “Others” represents a total of 73 other families.

The invasive plants that have been studied were generally diverse, with 34 total orders represented in the data set. Six orders (Asterales, Caryophyllales, Fabales, Lamiales, Poales, and Rosales) represented between 60% and 70% of all invasive plants studied within each time period (Figure 6), with the “Other” subcategory (representing 28 other orders of plants) making up the remainder. A total of 79 families were represented in the data set, with six families (Asteraceae, Brassicaceae, Caprifoliaceae, Fabaceae, Pinaceae, and Poaceae) making up between 50% and 60% of all invasive plants studied within each time period (Figure 7). The top 5 invasive plant species studied were *Alliaria petiolata* (Garlic mustard), *Berberis thunbergia* (Japanese barberry), *Bromus tectorum* (cheatgrass), *Lonicera maackii* (Amur honeysuckle), and *Microstegium vimineum* (Japanese stiltgrass) (Table 2), all of which are invasive in North America and/or Europe. These five species represented 74 studies, or about 16% of the total number of papers in the data set (Table 2).

**TABLE 2:** Top 5 most studied invasive plant species within studies published in *Biological Invasions* and *Neobiota* from 2009 to 2020

Species	No. of papers published in each time period				
	1999–2000	2004–2005	2009–2010	2014–2015	2019–2020
*Alliaria petiolata*	2	1	5	7	3
*Berberis thunbergii*	3	2	2	3	3
*Bromus tectorum*	1	2	4	6	4
*Lonicera maackii*	0	0	3	6	3
*Microstegium vimineum*	0	1	11	1	1

**FIGURE 8:**
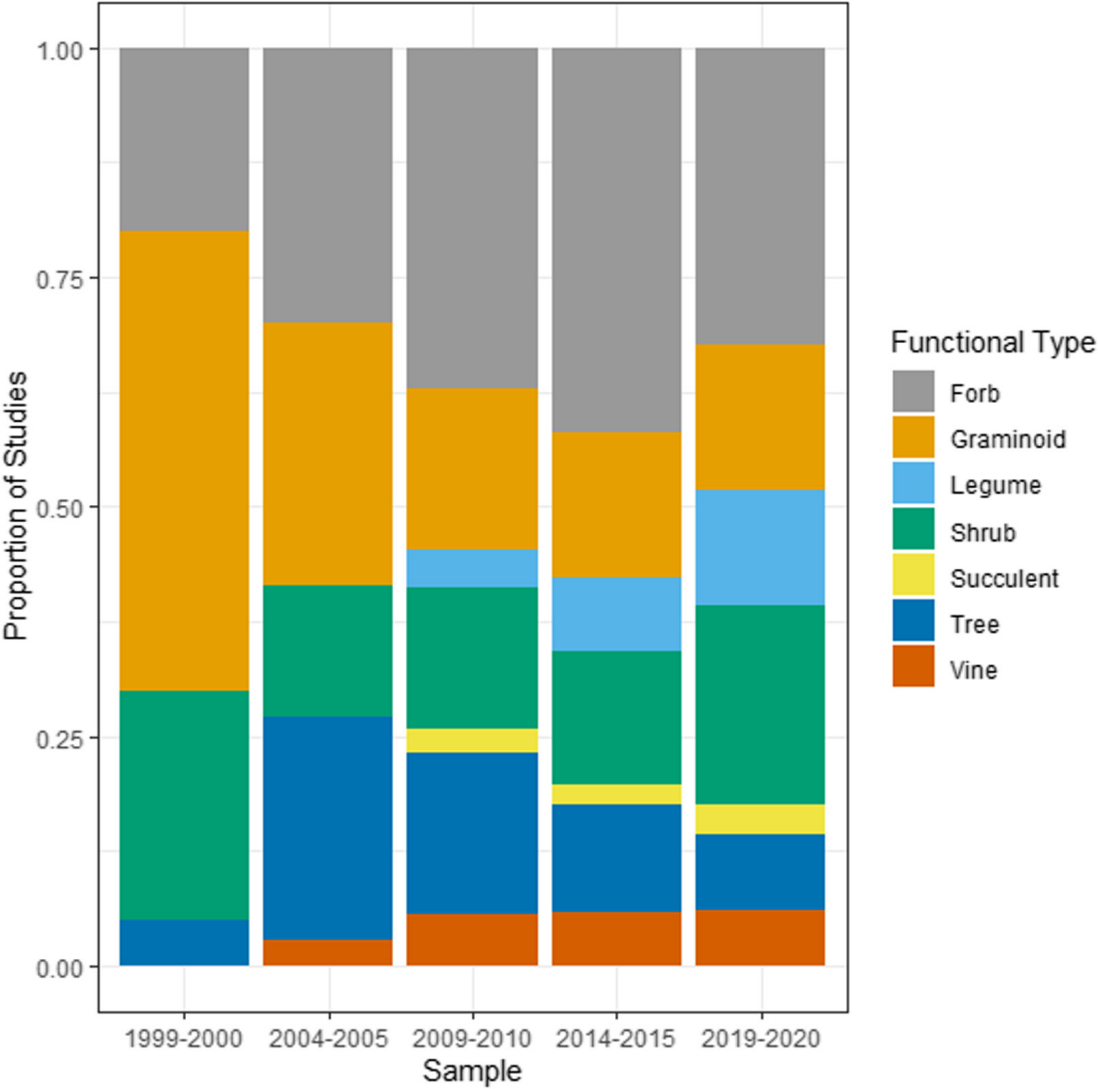
Invasive plant functional types studied within invasive plant studies published in *Biological Invasions* and *Neobiota* from 1999 to 2020, showing proportion of studies published.

The Astarales/Asteraceae (Daisies and related plants) and Poales/Poaceae (grasses, sedges, and reeds) are consistently among the most commonly studied invasive plants across time, and there is an increasing trend for scientists to study invasive legumes (i.e., Fabaceae) (*χ* ^2^ = 6.2, df = 1, *p* = .001) (Figure 7). These patterns are reflected in the data of invasive plant growth habits/functional types, which is consistently dominated by forbs, shrubs, and grasses (graminoids) and a more recent increase in the proportion of invasive legume studies (*χ* ^2^ = 19.4, df = 1, *p* < .001) (Figure 8). Of the other plant types, there was a distinct increase in number studies on invasive trees up until 2009–2010 data set, but with a decline thereafter.

### 3.4 Types of topics being studied

There are clear trends in the type of topics being studied in invasive plant papers across time (Table 3). There is decreasing interest in establishment‐type questions (i.e., “How did the invasive plant get/survive here?”) (*χ* ^2^ = 11.5, df = 1, *p* < .001), coupled with a nonstatistically significant increase in impact‐type questions (i.e., “What is it doing to invaded ecosystems?”) (*χ* ^2^ = 2.6, df = 1, *p* = .104). There is also a peak in genetic studies during the 2009–2015 time period. Finally, there is also a marginally statistically significant increase of interest in recent years (notably in 2019/2020) in plant physiological trait research (*χ* ^2^ = 3.2, df = 1, *p* = .076), which appears to be driven primarily by increased interest in the role of ecophysiological and functional traits in invasive plant success within invaded ecosystems.

**TABLE 3:** Questions asked within invasive plant studies published in *Biological Invasions* and *Neobiota* from 2009 to 2020

Question type	% of papers published in each time period				
	1999–2000	2004–2005	2009–2010	2014–2015	2019–2020
Establishment	42.11	31.58	25.63	11.57	17.18
Spread & dispersal	5.26	0.00	5.00	3.31	10.43
Plant traits (physiological)	10.53	18.42	11.25	11.57	20.86
Plant traits (genetic)	0.00	2.63	7.50	10.74	4.29
Impact (native communities)	15.79	13.16	27.50	26.45	25.15
Impact (soil/environment)	0.00	7.89	6.25	10.74	8.59
Control	26.32	21.05	7.50	22.31	9.20
Others	0.00	5.26	9.38	3.31	4.29

## 4 DISCUSSION

### 4.1 Geographical distribution

Characteristic of most fields of ecology (Cameron et al., 2019; Clarke et al., 2017), there is a large bias in invasive plant research at least within the two main journals exclusively devoted to invasion biology (*Biological Invasions* and *Neobiota*) toward wealthier countries, with early interest in invasive species coming largely from the regions of North America, Europe, Australia, and New Zealand. In particular, the region with the largest contribution across the 20‐year period of our analysis was North America. This is consistent with previous reports highlighting general geographical bias in the field invasion biology (Chong et al., 2021; Nuñez et al., 2022; Pyšek et al., 2008; Qiu & Chen, 2009). The top 5 most studied invasive plants within this 22‐year period are also all well‐known invasive plants in North America. This may reflect the greater amount of resources being allocated to invasion biology research in wealthier countries (Leimu & Koricheva, 2005; Pyšek et al., 2006), or greater recognition of the problems invasive plants can cause within these regions. As such, it is possible that high levels of socioeconomic development may indirectly facilitate plant invasions, via higher anthropogenic disturbances and habitat destruction (Essl et al., 2019).

While less wealthy regions are generally underrepresented in the data set used in our analysis, the amount of invasive plant research in some of these regions has shown increases over the past 20 years. In particular, middle‐income countries such as China, South Africa, and some in South America are clearly showing strong recent upticks in the number of invasive plant papers being published. These regions have notably increased funding toward scientific research in general, as well as toward invasion biology (Liu et al., 2012; van Wilgen, 2020), and there could also be an increasing awareness of the problems invasive plants can cause in these regions (Fuentes et al., 2010; Speziale & Lambertucci, 2010; van Wilgen & Wilson, 2018). This is an important trend in this field, as invasive plants are projected to become a greater threat in developing countries due to globalization (Chong et al., 2021; Seebens et al., 2015, 2021) and climate change (Dullinger et al., 2017), and a better understanding of the processes underlying plant invasions within these regions is crucial to understanding the ecology of plant invasions worldwide.

### 4.2 Climatic zone and ecosystem type

The vast majority of invasive plant studies across the 22‐year period of our analysis has been conducted in Mediterranean, subtropical, and temperate climatic zones. This is reflective of the geographical biases toward studies conducted in North American, European, and Australian regions, and these regions also account for most studies in arid climates (i.e., mostly in North American deserts) and cold climates (i.e., mostly alpine, subarctic, and boreal sites in North America and Europe). There is a conspicuous dearth of studies conducted in tropical climates, with only a recent uptick in 2019/2020. While this may represent logistical issues in studying tropical field sites, it also demonstrates the relatively unknown extent of plant invasions and limited research performed within the tropics (Chong et al., 2021). While some available literature proposes that tropical ecosystems, being highly biodiverse habitats, may not be as invasible (i.e., easily invaded) (Lonsdale, 1999; Rejmánek, 1996; Teo et al., 2003; but see Chong et al., 2021; Fine, 2002), the shortage of studies conducted in tropical ecosystems make it difficult to determine the extent to which invasive plants are an issue in the tropics. This is further complicated by the fact that many existing tropical studies on invasive plants have been conducted on tropical islands, notably the Hawaiian Islands (Chong et al., 2021; Weidlich et al., 2020), which are unrepresentative of most tropical landmasses and may have very different ecological characteristics to continental tropical ecosystems (Essl et al., 2019). Tropical systems are also facing increased and substantial ongoing anthropogenic threats and disturbances (Barlow et al., 2018; Fine, 2002; Gardner et al., 2009; Laurance, 2013), and the resulting plant invasions may have a time lag of several decades before becoming apparent through an “invasion debt” (Chong et al., 2021; Seebens et al., 2015).

In terms of ecosystem types, most invasive plant research over the 22 years of our analysis has consistently been conducted within forests and grasslands. This may reflect ease of access and logistics, or that grasslands and forests receive more funding for invasion research due to public interest and/or economic reasons such as asset protection (Hiatt et al., 2019). While the proportion of studies being conducted across different ecosystems have remained fairly constant over time, many understudied systems (such as urban/anthropogenic and desert systems) have received increased research interest in recent years, reflecting awareness that few ecosystem types or habitats are immune to plant invasion.

### 4.3 Plant taxonomic and functional identity

While there is a very large number of potentially invasive plant species, our analysis shows the vast majority of invasive plant research at least within the two journals devoted exclusively to invasion biology (*Biological Invasions* and *Neobiota*) has consistently focused on smaller plants such as forbs, shrubs, and grasses. Such plants often have traits that enable invasion, such as being easily dispersed and growing relatively quickly to maturity (Martín‐Forés et al., 2018). They are also easily studied by researchers—the same traits that make them good invasive plants also make them easy to manipulate and grow in field and greenhouse experiments. In contrast, there has been a decline in invasive tree studies since 2009/2010—the longer‐lived nature of trees may make them less amenable to short‐term experimental studies within the duration of conventional funding cycles.

There has also been a steady increase in studies in nitrogen‐fixing plants (i.e., legumes within Fabaceae) over the 22 years covered by our analysis. Nitrogen fixation is well recognized as a major factor enabling invasion success, and nitrogen‐fixing species feature disproportionately in many invasive floras (Daehler, 1998; Richardson et al., 2000). These species are also of interest to researchers because of their major role in driving plant–soil feedbacks (van der Putten et al., 2013; Wardle et al., 2004) and their transformative effect on soil nutrient cycles and ecosystem processes (Vitousek et al., 1987; Vitousek & Walker, 1989; Wardle & Peltzer, 2017). In particular, nitrogen fixers do especially well in N‐limited and P‐replete systems (Smith, 1992; Zheng et al., 2016), which are common in early successional and heavily disturbed systems (Craft, 1996; Smith et al., 2000), and invasive nitrogen fixers may therefore be well adapted to invade and dominate early in successional processes.

### 4.4 Types of topics being studied

Our analysis shows that over the past two decades, there has been a relative decline in the proportion of studies that have addressed questions about the establishment phase of the invasion process, and an increase in the proportion those on the ecological impacts of invasive species. As such, there is growing awareness that invasive plants frequently have negative impacts on native species and community structure, and the abiotic components of native ecosystems (e.g., soil properties, biogeochemical cycles). Research over the past 20–25 years has also shown how plant species differences might impact ecosystem properties, soil properties, and plant–soil feedbacks (van der Putten et al., 2013; Wardle et al., 2004; Wardle & Peltzer, 2017), and that invasive and native species may differ in their abiotic impacts, therefore impacting (and in some cases transforming) ecosystems.

Changes in research foci over the past 20 years in our analysis also may reflect changes over time in what are perceived to be “hot” research topics across ecology in general. The life sciences boom in the 2000s–2010s was accompanied by a rapidly growing interest in applying genetic methods to answer ecological questions (Ohniwa et al., 2010; Ungerer et al., 2008); in our analysis this is likely to have caused the peak in genetic research on invasive plants in within these two nongenetics‐focused journals 2014–2015 before tapering off. Plant functional and ecophysiological traits have become a “hot” topic in plant ecology research in recent years (Cornwell et al., 2008; de Bello et al., 2010; Diaz et al., 2004; Pantel et al., 2017), and this is accompanied by a continual increase in trait‐focused studies in invasion biology over the past 20 years in our analysis. This literature is often connected to the idea that invasive plants have more acquisitive traits than do native species (Mathakutha et al., 2019; van Kleunen et al., 2010) that may impact on ecosystem processes including those in the soil (Vilà et al., 2011; Wardle & Peltzer, 2017), and that they possess phenological traits that enable faster, more widespread dispersal (Martín‐Forés et al., 2018).

## 5 CONCLUSIONS

As a consequence of the geographical, climactic, and ecosystem biases in the ecological literature in general, our current models, concepts, and understanding of the dynamics of plant invasions are still largely biased by temperate grassland and forest ecosystems in the Americas, Europe, and the Australian regions, and these may not necessarily apply to other types of ecosystems or climates. Our understanding of invasive plant biology may also be biased toward smaller, and often herbaceous plants. However, these biases are partially offset by recent increasing plant invasion research in understudied systems, as well as increasing awareness that plant invasion is heavily affected by their growth types, physiological traits, and soil interactions. Invasive plant research is also not immune to being influenced by whatever happens to be the “hot” research topic of the day. As such, researchers in this field need to be cautious to not be overly influenced by prevailing paradigms caused by biases in the literature.

The field of invasion biology will only become more important over time, and focusing invasive plant research on understudied and threatened ecosystems will allow us to develop a more holistic understanding of the ecological dynamics of invasive plants. In particular, given the outsize importance of the tropics to global biodiversity, the threats that their ecosystems face, and the dearth of studies in tropical regions, it is of critical importance that more invasive plant research is conducted within the tropics to better understand the threats they pose there. This study looked only at the existence of biases over time within invasive plant research, and this approach does not fully cover the many structural issues (such as logistics, lack of funding, and other reasons) that may cause such biases and hinder research in the tropics and other understudied areas (see Chong et al., 2021). It is therefore critical that the organizations and people (such as researchers, journals, and funding organizations) that enable scientific research understand these limitations, as well as work to reduce or eliminate the hurdles involved in carrying out such research in underrepresented regions. We conclude that a greater presence of underrepresented regions, ecosystems, and taxa in the literature is also essential in ensuring that the conclusions drawn from literature syntheses and meta‐analysis and theory about invasion biology will not only be applicable or true for a subset of the world's ecosystems and invasive biota.

## AUTHOR CONTRIBUTIONS

**Kwek Yan Chong:** Conceptualization (equal); formal analysis (supporting); investigation (equal); methodology (equal); software (equal); supervision (equal); validation (equal); writing – review and editing (equal). **Shawn K. Y. Lum:** Conceptualization (equal); investigation (equal); methodology (equal); supervision (equal); validation (equal); visualization (equal); writing – review and editing (equal). **David A. Wardle:** Conceptualization (equal); formal analysis (supporting); investigation (supporting); methodology (equal); project administration (lead); supervision (lead); validation (equal); visualization (supporting); writing – review and editing (equal). **Jing Hua Chiu:** Conceptualization (equal); data curation (lead); formal analysis (lead); investigation (equal); methodology (equal); software (lead); visualization (lead); writing – original draft (lead); writing – review and editing (equal).

## ACKNOWLEDGEMENTS

We would like to thank two anonymous reviewers for their helpful comments and suggestions for our paper.

## Data Availability

The data that support the findings of this study are available from the corresponding author upon reasonable request (upon acceptance the raw data will be deposited in Dryad and the https://doi.org/10.5061/dryad.wdbrv15ss. Data is currently being uploaded to the above DOI on Dryad).
